# Dietary Polyphenols as a Protection against Cognitive Decline: Evidence from Animal Experiments; Mechanisms and Limitations

**DOI:** 10.3390/antiox12051054

**Published:** 2023-05-05

**Authors:** Ruth Naomi, Muhammad Dain Yazid, Soo Huat Teoh, Santhra Segaran Balan, Halim Shariff, Jaya Kumar, Hasnah Bahari, Hashim Embong

**Affiliations:** 1 Department of Human Anatomy, Faculty of Medicine and Health Sciences, Universiti Putra Malaysia, Serdang 43400, Malaysia; gs60018@student.upm.edu.my; 2 Centre for Tissue Engineering and Regenerative Medicine (CTERM), Universiti Kebangsaan Malaysia, Kuala Lumpur 56000, Malaysia; dain@ukm.edu.my; 3 Advanced Medical and Dental Institute, Universiti Sains Malaysia, Penang 13200, Malaysia; soohuat@usm.my; 4 Department of Diagnostic and Allied Health Sciences, Faculty of Health and Life Sciences, Management and Science University, Shah Alam 40100, Malaysia; santhra@msu.edu.my; 5 Faculty of Health Sciences, University Technology Mara (UITM) Pulau Pinang, Bertam Campus, Kepala Batas 13200, Malaysia; drhalim_shariff@msu.edu.my; 6 Department of Physiology, Faculty of Medicine, Universiti Kebangsaan Malaysia, Kuala Lumpur 56000, Malaysia; jayakumar@ukm.edu.my; 7 Department of Emergency Medicine, Faculty of Medicine, Universiti Kebangsaan Malaysia, Kuala Lumpur 56000, Malaysia

**Keywords:** dietary polyphenols, neurodegeneration, mechanisms of neuroprotection, bioavailability, safety efficacy

## Abstract

Emerging evidence suggests that cognitive impairments may result from various factors, such as neuroinflammation, oxidative stress, mitochondrial damage, impaired neurogenesis, synaptic plasticity, blood–brain barrier (BBB) disruption, amyloid β protein (Aβ) deposition, and gut dysbiosis. Meanwhile, dietary polyphenol intake in a recommended dosage has been suggested to reverse cognitive dysfunction via various pathways. However, excessive intake of polyphenols could trigger unwanted adverse effects. Thus, this review aims to outline possible causes of cognitive impairments and how polyphenols alleviate memory loss via various pathways based on in vivo experimental studies. Thus, to identify potentially relevant articles, the keywords (1) nutritional polyphenol intervention NOT medicine AND neuron growth OR (2) dietary polyphenol AND neurogenesis AND memory impairment OR (3) polyphenol AND neuron regeneration AND memory deterioration (Boolean operators) were used in the Nature, PubMed, Scopus, and Wiley online libraries. Based on the inclusion and exclusion criteria, 36 research papers were selected to be further reviewed. The outcome of all the studies included supports the statement of appropriate dosage by taking into consideration gender differences, underlying conditions, lifestyle, and causative factors for cognitive decline, which will significantly boost memory power. Therefore, this review recapitulates the possible causes of cognitive decline, the mechanism of polyphenols involving various signaling pathways in modulating the memory, gut dysbiosis, endogenous antioxidants, bioavailability, dosage, and safety efficacy of polyphenols. Hence, this review is expected to provide a basic understanding of therapeutic development for cognitive impairments in the future.

## 1. Introduction

Neurological disorders are becoming a global burden, and it is estimated that there might be an upsurge in the prevalence of neurological disorders, especially in low- and middle-income countries, over the next 10 years. Since the brain is the most vulnerable organ and the main target for neurological conditions, severe disability and poor quality of life are often seen in people with neurological conditions. One of the common manifestations of neurological conditions is cognitive decline [1]. Neurological disorders have been classified as one of the most common causes of morbidity and are ranked second in the global causes of morbidity [2]. Interventions to manage or treat neurological conditions can be very challenging due to the presence of the highly selective semi-permeable membrane known as the blood–brain barrier (BBB) and its unique capability to shield the brain from xenobiotics. Although conventional therapeutics are effective, their therapeutic efficacy is still below the optimum level. Therefore, there is currently a research focus on developing methods deliver chemical across the BBB [3]. Hence, natural products are gaining more popularity as preventive medicine and therapeutics in the treatment of neurological disorders. As a result, the use of natural products has gained the attention of researchers—especially natural extracts that have antioxidant properties [4]. Polyphenols are said to be effective in preventing neurodegeneration and neuroinflammation, thereby preventing cognitive decline even in very small dosages [5]. This may be due to the ability of certain polyphenols—such as epigallocatechin, daidzein, genistein, equol, and nobiletin—to be highly permeable to the BBB [6]. Thus, this review addresses the various factors that contribute to cognitive impairment; the possible mechanisms by which polyphenols alleviate memory loss, including reducing neuroinflammation and oxidative stress, improving mitochondrial function and synaptic plasticity, modulating gut microbiota; and reducing Aβ deposition.

This review highlights the importance of appropriate dosage of polyphenols, taking into account individual factors, such as gender, and underlying conditions to avoid adverse effects, such as gastrointestinal discomfort, liver toxicity, and interference with nutrient absorption. The study concludes that while the animal experiments provide a strong foundation for the therapeutic potential of dietary polyphenols in cognitive decline, more research is needed to determine the optimal dosage and long-term effects of polyphenol intake in humans. The study also emphasizes the importance of considering individual factors when recommending polyphenol intake for memory enhancement. Overall, this review provides an overview of the potential therapeutic benefits of dietary polyphenols for cognitive impairment and highlights the need for further research in this area. The study’s goals and objectives are to review the evidence for the potential of dietary polyphenols to protect against cognitive decline and to identify the mechanisms by which polyphenols alleviate memory loss via various pathways based on in vivo experimental studies. The study is relevant and novel in that it provides a thorough review of the current literature on this topic and highlights the importance of appropriate dosage and individualized recommendations for polyphenol intake to achieve maximum benefits while avoiding adverse effects.

## 2. Polyphenols

**Figure 1:**
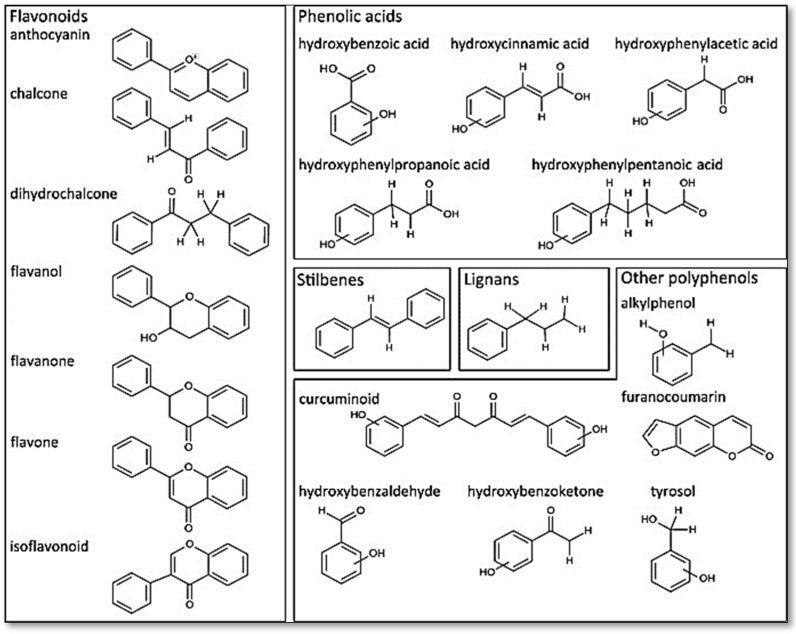
Distinct substructure of polyphenols. Figure reused under the permission granted by http://creativecommons.org/licenses/by/4.0/ (accessed on 27 April 2023) [10].

The polyphenols are a large and diverse group of naturally occurring compounds found in plants and are characterized by the presence of multiple phenol rings. They are widely distributed in the plant kingdom and are believed to play a key role in protecting plants against ultraviolet radiation, pathogens, and oxidative stress. To date, 8000 phenolic molecules have been identified [7]. Sources of polyphenols include fruit, vegetables, whole grains, nuts, seeds, and herbs. Polyphenols can be classified into two categories based on their chemical structure: flavonoids and non-flavonoids (phenolic acids, stilbenes, lignans, and others). They can be divided into many sub-classes depending on the number of phenol units within their molecular structures, their substituent groups, and/or the linkage type between phenol units [8]. The presence of phenolic compounds in plants can occur in both free and conjugated forms, with one or more sugar residues joined by β -glycosidic bonds to a hydroxyl group or an aromatic ring’s carbon atom [9]. Figure 1 provides some examples of distinct structures of polyphenols.

### 2.1. Flavonoids

Flavonoids such as flavonols (found in onions, kale, and tea), flavanones (found in citrus fruits), isoflavaones (found in onions, kale, and tea), flavones, flavan-3-ols (found in tea, cocoa, and some fruits), and anthocyanins are among the most abundant polyphenols available in plants or food and are the most commonly studied. The great variability of the molecules (about 6000 distinct structures) comes from modifications of the core structures, such as by hydroxylation, methylation, glycosylation, and acylation, among others [11]. These modifications can occur at different positions of the rings, leading to a wide variety of flavonoid compounds with different biological activities and functions. Flavonoids can be found as glycosides or aglycones despite the fact that their basic structures are aglycones (the nonsugar part of the corresponding glycoside). All flavonoids share the same basic structure of diphenyl propanes (C_6_-C_3_-C_6_); they are composed of two aromatic rings (A and B) joined by a three-carbon bridge (C) and contain a carbonyl group (C=O) at position 1 in ring C, forming a central pyrane ring [12].

### 2.2. Flavanones

Flavonones are a type of compound derived from a chalcone-like compound and use flavan-4-ol as a substrate. The heterocyclic ring in flavanones or dihydroxyflavones (hesperetin, naringenin, eridictyol, sylibin, isosakuratenin), has a saturated three-carbon chain without a hydroxyl group at the C_3_ position, which makes it different from flavonols and flavones. In addition, due to their distinct substitution patterns, flavanones are characterized by a significant number of substituted derivatives, such as prenylated flavanones and benzylated flavanones [13]. Flavanones are recognized as important phytochemicals and are mainly found in high concentrations in citrus fruits, such as lemons (e.g., eriodictyol) and oranges (e.g., hesperidin), and most of these compounds are found in aglycone forms [14].

### 2.3. Flavones

Flavones differ from other flavonoids since they contain a double bond between the atoms C_2_ and C_3_ and a ketone group at the atom C_4_. Some of the flavones include luteolin, apigenin, chrysin, baicalein, tangeritin, diosmetin, orientin, and scoparin. They are synthesized from flavanones utilizing flavone synthase, which catalyzes the oxidation of C_2_ and C_3_ atoms and the formation of the double bond between them. A high degree of chemical diversity is produced by various modifications of the flavone backbone, which leads to a variety of biological actions. Flavones from plants are typically conjugated as 7-O-glycosides via glycosylation, which can enhance the solubility and biological activity of flavone [15,16]

### 2.4. Isoflavones

Isoflavones are a group of isoflavonoids primarily found in legumes, such as lentils, fava beans, and soybeans, which are the main source of isoflavones in the human diet. Isoflavones resemble estrogens structurally, especially 17-estradiol. Isoflavones resemble estradiol in that they have hydroxyl groups in the C_7_ and C_4_ positions and have ring B attached to ring C at the C_3_ position of the latter ring. Isoflavones are known for their ability to mimic the effects of estrogen in the body by binding to estrogen receptors, and as such, they are sometimes referred to as phytoestrogens. Some of the common isoflavones include genistein, daidzein, and glycitein [17,18].

### 2.5. Flavonols

Flavonols (dihydroflavonols) are the 3-hydroxy derivatives of flavanones that are synthesized by flavonol synthase. They consist double bond between C_2_ and C_3_ and oxygen (a ketone group) in C_4_ and particularly with flavones by the presence of a hydroxyl group in the C_3_ position [19]. The main sources of flavanols are cocoa, followed by dark chocolate, berries, strawberry, and nuts. Some foods or plants that are rich in flavonols may also contain significant amounts of catechins and proanthocyanidins [20]. However, the concentration of these compounds can vary greatly depending on the specific food or plant source. Some commonly studied compounds in this group are myricetin, kaempferol, and quercetin [21].

### 2.6. Phenolic Acid

Phenolic acids are a type of organic compound that is widely distributed in plants. They can be found in many vegetables and fruits (caffeic acid), berries and nuts (ellagic acid), and coffee and some fruit (chlorogenic acid). They are derived from benzoic acid or cinnamic acid and are characterized by the presence of one or more hydroxyl groups (−OH) attached to an aromatic ring. The structure of phenolic acids can vary depending on the number and position of the hydroxyl groups on the ring. Phenolic acids can be classified into two main classes based on their structure which are hydroxybenzoic acids (HBAs) and hydroxycinnamic acids (HCAs). HBAs have a benzoic acid backbone and one or more hydroxyl groups (−OH) attached to them, and examples of HBAs include gallic acid, protocatechuic acid, vanillic acid, and syringic acid. They are found either as free acids or in their conjugated forms (glycosides or esters). Meanwhile, a cinnamic acid backbone with one or more hydroxyl groups (-OH) attached to it is present in HCAs. Examples of hydroxycinnamic acids include caffeic acid, ferulic acid, and sinapic acid [22].

### 2.7. Lignans

Lignans consist of two propylbenzene units (C_6_-C_3_) linked together between the β-position in C_8_ of the propane side chains. The C_9_ and C_9_’ positions of lignans are substituted in different patterns, resulting in a wide range of different structural forms [23,24]. Lignans are found in legumes, seeds, and vegetable oils; they are mainly found in their free forms, while the glycosylated structure is not abundant [25]. The most commonly studied lignans are pinoresinol, secoisolariciresinol, medioresinol, lariciresinol, and syringaresinol [26].

### 2.8. Stilbenes

Stilbenes (1,2-diarylethenes) are a group of organic compounds that are composed of two benzene rings connected by a two-carbon ethylene bridge (−CH=CH−). The simplest stilbene is trans-stilbene, which has two phenyl groups attached to each end of the ethylene bridge. Stilbenes can exist as a glycosylated compound (major) or freeform (minor). This secondary metabolite is derived from the phenylpropanoid pathway commonly found in plants such as peanuts, grapes, and some berries. One of the most well-known stilbenes is resveratrol. Resveratrol (cis and trans), which is highly concentrated and naturally present in the fresh skin of red grapes, is identified as a main compound of stilbenes [25,27].

## 3. Cognitive Decline

Several factors contribute to cognitive decline. Some of the common causes include neuroinflammation, oxidative stress, synaptic plasticity, disruptions in the blood–brain barrier (BBB), mitochondrial damage, β-amyloid (Aβ) deposition, impaired neurogenesis, and altered gut–brain axis. Thus, this section briefly explains the possible causes that could lead to cognitive impairments.

### 3.1. Neuroinflammation

A key cause of cognitive decline and neurodegenerative disorders, such as Alzheimer’s disease, Parkinson’s disease, Huntington’s disease, multiple sclerosis, and amyotrophic lateral sclerosis, is neuroinflammation. Almost all diseases of the central nervous system exhibit neuroinflammation, which is now widely acknowledged as a potential mediator of cognitive decline. Neurodegeneration and advanced age both increase the levels of systemic inflammation. For example, age-related changes in neuroinflammatory responses, such as glial activation and increased production of proinflammatory cytokines, can contribute to abnormal neuronal signaling and cognitive decline. However, it is also possible that abnormal neuronal signaling may trigger the release of proinflammatory cytokines and activate glial cells, leading to a neuroinflammatory response. Furthermore, other factors, such as activated microglia, astrocytes, inflammasomes, and bad cholesterol, can also contribute to the deterioration of the central nervous system microenvironment and exacerbate neuroinflammation and cognitive decline [28,29,30].

### 3.2. Oxidative Stress

Studies on cells, biochemistry, and molecules have revealed close connections between oxidative stress and cognitive decline with age and age-related neurological disorders. Reduced repair and oxidative damage to nuclear and mitochondrial DNA are symptoms of brain aging. The antioxidant pathways are complex defense mechanisms that guard against oxidative damage to the human body. They include enzymes such as catalase (CAT), superoxide dismutase (SOD), glutathione peroxidase (GPx), and many other nonenzymatic antioxidants that are either endogenous, such as glutathione (GSH), or dietary, such as vitamins A, C, and E and carotenoids. Thus, the degeneration of neurons, which is typically evident in brain illnesses, is caused by the accumulation of oxidative stress damage from oxidized proteins, glycated products, and lipid peroxidation. Cerebrovascular disorders are known to cause cognitive decline and are defined as vascular lesions [31,32].

### 3.3. Mitochondrial Damage

The mitochondria are the cell’s power plants, producing most of the adenosine triphosphate (ATP) through oxidative phosphorylation [33]. Among all cell types, neurons are among the most intensely energy- (ATP-) consuming cells due to their high metabolic demands. This is because the maintenance of ionic gradients across the neuronal cell membrane and the firing of action potentials require a substantial amount of ATP [34]. Additionally, another reason could be due to the need to preserve the ionic gradients necessary for continuing electrophysiological activity, neurotransmission, and temporary synaptic plasticity [35]. For example, low basal cytoplasmic concentrations and storage in calcium (Ca^2+^) release organelles are necessary for efficient intracellular Ca^2+^ signaling and are maintained by ATP-dependent mechanisms. Moreover, damaged mitochondria are important sites for producing free radicals and can cause apoptosis by releasing cytochrome c into the cytoplasm. Hence, even a slight decline in mitochondrial function may cause harm to neurons. The “mitochondrial theory of aging” proposes that mitochondria accumulate oxidative damage as reparative effectiveness declines, leading to inadequate cellular bioenergetics. In turn, metabolic disturbance results in altered synaptic plasticity, which impairs cognitive function, decreased neural resistance to other types of damage, and subsequent neurodegeneration. By leaking electrons throughout the electron transport cascade for oxidative phosphorylation, mitochondria produce free radicals that cause oxidative stress and impair cellular signaling and metabolic processes [36,37].

### 3.4. Synaptic Plasticity

Modifications to the morphology and function of synapses control synaptic plasticity, which is directly related to memory and learning processes. The two main manifestations of synaptic plasticity are long-term potentiation and long-term depression. The dendritic membrane contains projecting, tiny, highly dynamic structures called dendritic spines. These spines contain signal transduction molecules, scaffolding proteins, ion channels, cytoskeleton components, and postsynaptic density, which are mostly composed of α-amino-3-hydroxy-5-methyl-4-isoxazolepropionic acid receptors (AMPAR) and NMDAR [38]. Synaptic plasticity is mediated through structural alterations such as spine elongation and contraction, shape variations, spine distribution/density, and other functions. In the hippocampus and neocortex, abnormal synaptic structure/morphology and decreased spine density are early occurrences and significant changes that are associated with cognitive problems. Such changes in synaptic activity may enhance or impair rhythmic electrical activity, which in turn affects plasticity, demonstrating a two-way interaction [39].

### 3.5. Impaired Neurogenesis

The process through which new neurons are created in the brain is known as neurogenesis. Therefore, impaired neurogenesis could result in cognitive impairments. Studies show an activity-sensing property of hippocampal neural progenitor cells via Ca_v_1.2/1.3 (L-type) Ca^2+^ channels and N-methyl-D-aspartate (NMDA) receptors, suggesting that excitation of the local neural network may regulate the neurogenic process. Neurogenesis modulates hippocampal network activity to enable memory storage at different levels [40]. In fact, during hippocampus neuronal development, such activity-dependent responses may aid in the creation of new memories and the erasure of old ones [41]. Progressive memory impairment is associated with degeneration of the hippocampus. The dentate gyrus of the hippocampus is a region critical for learning and memory functions. This will interpret the neurogenesis process and cause balance at brain hemostasis. When the neurogenesis process is not able to process, it may cause cognitive decline [42,43].

### 3.6. Blood–Brain Barrier Disruption

The BBB is essential for sustaining the neural tissue’s unique milieu. It facilitates communication while separating the brain parenchyma from the peripheral circulation system [44]. The neurovascular unit, which includes pericytes, smooth muscle cells, astrocytes, microglia, oligodendroglia, and neurons, forms the continuous non-fenestrated endothelial cells that make up the BBB at the cellular level [45]. The BBB is selectively permeable, meaning it allows essential nutrients to enter the brain while preventing harmful substances and toxins from crossing into the brain [44]. During the natural aging process, the BBB undergoes a range of structural and functional changes, causing the BBB to break down. Alterations in the composition and organization of the endothelial cell junctions, changes in the number and morphology of pericytes and astrocyte end-feet, and increases in oxidative stress and inflammation are some of the changes that can occur in the natural aging process in the BBB. Resultantly, the permeability of the BBB will increase, allowing potentially harmful substances to cross the BBB and induce damage. The BBB breaks down in the brain, which causes an increase in BBB permeability and a decrease in cerebral blood flow. As aging occurs, the capacity of neovascularization declines, as does the capillary density of the brain vasculature. According to research, the first occurrence of BBB breakdown in the aging hippocampus is what may cause cognitive impairments. The growing dysfunctional state of the brain endothelium is associated with abnormal BBB alterations [46,47].

Along with neurodegenerative diseases, such as Alzheimer’s, Parkinson, amyotrophic lateral sclerosis, multiple sclerosis, and Huntington’s disease [48], virus infection (ex. herpes simplex virus encephalitis) [49] and brain tumors [50] could also disrupt the normal structure of the BBB. In multiple sclerosis, the immune system attacks and damages the myelin sheath that surrounds nerve fibers in the brain and spinal cord, causing inflammation and disruption of the BBB, which allows the immune cells and antibodies to enter the brain [51]. Meanwhile, in the case of herpes simplex virus (HSV) encephalitis, the virus can directly infect and damage the BBB, leading to vascular brain edema, hemorrhage, and leukocyte infiltration, thereby permitting the entrance of viruses, immune cells, and cytokines into the brain parenchyma [49]. Similarly, in the vicinity of brain tumors, the integrity of BBB is disrupted due to the uncontrolled division of tumor cells and metastases and the presence of inflammation, as the tumor cells themselves produce inflammatory molecules [50].

### 3.7. β-Amyloid (Aβ) Deposition

A protein fragment called beta-amyloid (*Aβ*) is more frequently found in the brain as sticky, starch-like plaques [52]. The amyloid precursor proteins are cut into beta-amyloid protein fragments. According to a widely held belief, these protein pieces are broken down and discarded in a brain that is functioning ideally, but in some circumstances, this does not happen, and the protein fragments instead build up in the brain to form plaques. Instead of a failure to clear these products, the plaques are caused by an overproduction of amyloid precursor protein, which starts the cytotoxic effects. The result could be cognitive deterioration [52].

### 3.8. Gut–Brain Axis

The gut–brain axis, a bidirectional communication network that connects the enteric and the brain, is recognized to have a close connection with the central nervous system. This involves the ability of the gut microbiota to produce and release neurotransmitters and neuroactive metabolites that can affect brain functioning. For instance, a low level of *Bifidobacterium infantis* resulted in decreased production of serotonin and a high concentration of tryptophan in the plasma [53]. A marked decrease in serotonin in the neurons is positively associated with an increased level of cortical Aβ deposition and cognitive impairment [54]. Conversely, the gut microbial compositions of *Lactobacillus plantarum*, *Lactobacillus brevis*, *Bifidobacterium adolescentis*, *Bifidobacterium angulatum*, and *Bifidobacterium dentium* are known for their ability to produce gamma-aminobutyric acid (GABA) [55]. As an inhibitory neurotransmitter, GABA can control neuronal activity, reduce postsynaptic transmission hyperactivity, and regulate neural firing in the hippocampal [56]. Similarly, gut dysbiosis and disturbances to the gut–brain axis have a causal relationship with the development of neurological conditions, specifically cognitive performance [57].

## 4. Materials and Methods

### 4.1. Search Strategy

The search for potentially relevant articles for this review was done based on the Preferred Reporting Items for Systematic Reviews and Meta-Analyses (PRISMA) described elsewhere [58]. Boolean operators were used to identify the keywords for article searches. The selected keywords included (1) nutritional polyphenol intervention NOT medicine AND neuron growth OR (2) dietary polyphenol AND neurogenesis AND memory impairment OR (3) polyphenol AND neuron regeneration AND memory deterioration. The literature search was conducted from January 2013 to January 2023, thereby limiting the article search to the past 10 years of publication.

### 4.2. Inclusion Criteria

Only animal studies (in vivo) were considered to be included in this review. All articles published in the English language, within 10 years of publication, and with full-text accessibility were selected to be further screened. The selected articles must contain all of the following information: (1) animal model used; (2) intervention details, such as dosage duration, type, and method; (3) behavioral test used; and (4) findings focusing on cognition.

### 4.3. Exclusion Criteria

All secondary articles, thesis dissertations, proceedings, patents, and case reports were excluded from being further reviewed. Those articles that were written in any other language other than English and articles that contained inadequate details as described in the inclusion criteria were automatically rejected from being further reviewed. Any articles that focused on in vitro, ex vivo, or human trials (prospective or retrospective) were also excluded.

### 4.4. Data Extraction

Initial data extraction was performed by 4 reviewers independently (R.N., J.K., S.S.B., S.H.T., and H.B) by screening the title, abstract, and full text. All articles that meet the inclusion criteria were selected to be included in the review. Upon agreement, a standardized data extraction sheet was created in which all of the reviewers extract data based on the selected article independently. The data extraction table is shown in Table 1. Any disagreements during data extraction were resolved by discussing with the fifth reviewer (H.S). 

## 5. Results

### 5.1. Literature Search and Article Selection

**Figure 2:**
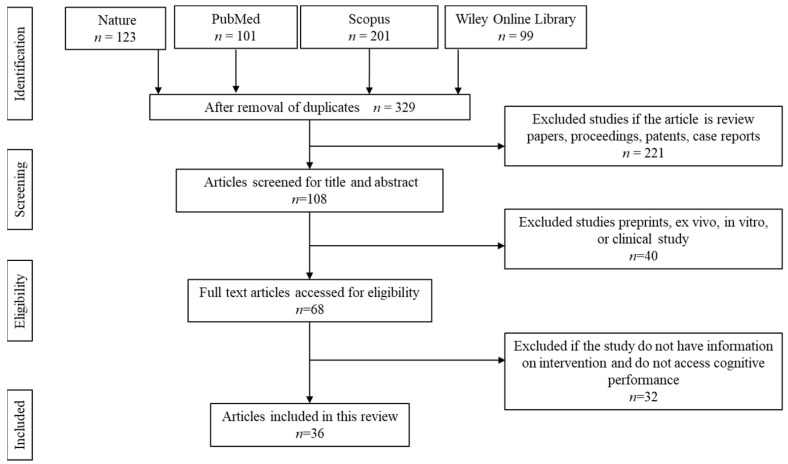
Article screening and selection.

The article selection and screening processes were conducted as shown in Figure 2. The initial screening resulted in a total of 524 articles. About 195 articles were removed owing to duplication. Of the remaining 329 articles, 221 articles were removed due to being secondary literature, case reports, proceedings, and patents. Another 40 articles were removed because the articles focused on in vitro, ex vivo, retrospective, or prospective studies. The remaining 68 articles were screened and accessed for full-text eligibility. From there, 32 articles were removed because the articles did not specify the details on the dose, duration, and type of intervention or if the study did not assess cognitive performance.

### 5.2. Mechanism of Polyphenols Alleviating Cognitive Decline

In vivo studies conducted for the past 10 years show that numerous incidents can cause cognitive decline as summarized in Table 1. Some of the pathological changes that induce cognitive impairments are neuroinflammation [59,71], oxidative stress [60,63,64,66,77], mitochondrial damage, impaired neurogenesis, disrupted blood–brain barrier (BBB) [92], and Aβ deposition [61,62,67,89,91]. In this context, polyphenols have proven to ameliorate cognitive decline and reverse pathological changes that occur in neurodegenerative diseases. For instance, dietary intake of polyphenol-rich fruits, such as apples (Ralls) [62], Prunus Salicina [67], Vitis vinifera [69], and Boswellia serrata gum [77] can reverse neuropathological changes associated with cognitive impairments in Alzheimer’s disease. Polyphenol intake is positively associated with cognitive performance improvements regardless of underlying conditions. One of the main reasons for this could be the ability of the polyphenols to cross the BBB and exhibit their effects. Since the BBB is selectively permeable, the relatively small size of the polyphenols enables them to cross the BBB and reach the targeted part of the brain (neurons) to exhibit their neuroprotective effects [96]. Polyphenols, being strong natural antioxidants, can neutralize excessive free radicals in the brain, thereby suppressing the activity of ROS. Thus, this section explains the mechanism of polyphenols in different pathways involved in cognition.

Polyphenols are proven to suppress neuroinflammation by suppressing proinflammatory factors, such as IL-1β, IL-6, and TNF-α, in the cerebral cortex and hippocampus. Microglial activation in neurotoxic conditions (M1) may stimulate the excessive release of proinflammatory cytokines, nitric oxides, and ROS, which may further activate the NLRP3 inflammasome signaling pathway [97]. The aggregation of Aβ is one of the causes of NLRP3 pathway activation. In such condition, upon activation NLRP3 will bind to the pyrin domain (death fold protein) and stimulates protease pro-caspase-1 to configure NLRP3 inflammasome, thereby pro-caspase-1 will become activated which is now known as caspase-1. Thus, caspase-1 is now free to bind with the inactive forms of proinflammatory cytokines and may induce the release of uncontrolled proinflammatory cytokines [98]. The strong proinflammatory signals may further induce pyroptosis in the neurons [99]. Hippocampal pyroptosis is one of the primary causes of cognitive decline in neurodegenerative diseases [100]. Conversely, Aβ may cleave to toll-like receptor 4 (TLR4) and induce microglial activation and excessive generation of cytokines. This may inhibit the microglial phagocytic process, causing the toxic form of Aβ (Aβ_1–42_) to increase, eventually leading to neuroinflammation [101]. Meanwhile, polyphenol compounds such as gallic acid and Salvianolic acid B can block the action of the NLRP3 inflammasome signaling pathway directly [102]. Several other polyphenols, such as curcumin, can inhibit the NLRP3 activity by blocking the action of the TLR-MyD88-NF-κB pathway, thereby preventing neuronal apoptosis [103,104]. In this, 100 mg/kg of curcumin is enough to inhibit TLR4-mediated proinflammatory cytokine release and repress microglial activation [104]. Another study shows that polyphenol (Catechin) intake could downregulate p38MAPK and NF-κBp65 signaling via the inhibition of TLR2, which may decrease the proinflammatory mediators and its signal transduction pathway [105].

Besides, the ability to neutralize free radicals is another way polyphenols exhibit neuroprotective effects. High levels of free radicals can be inferred from suppressed levels of endogenous antioxidants, including SOD, CAT, GPx, and GSH. In this, polyphenolic compound, such as Chlorogenic acid [88] and 5-Caffeoylquinic acid [89], is known to be an excellent free radical scavenger. This is achieved through the activation of Akt phosphorylation. Phosphorylated Akt in turn may inhibit the activity of GSK-3β, causing the level of cyclin D1 to increase, thereby modulating the cell cycle [106] and promoting neurogenesis by initiating neuronal differentiation [107]. Aside from this, caffeoylquinic acids protect hippocampal neurons from hydrogen-peroxide-induced oxidative stress by upregulation of nicotinamide adenine dinucleotide phosphate (NADPH) [108]. An alternative method of using 5-Caffeoylquinic to neutralize free radicals is decreasing the level of lipid peroxidation in the hippocampus, thereby restoring cognitive performance [109]. A high level of ROS may mediate lipid peroxidation, which may cause neuronal damage. In the case of neurological disorders such as Alzheimer’s, lipid peroxidation may induce accumulation of Aβ, while in Parkinson’s, it may induce the localization of 4-hydroxy-2-nonenal within the Lewy bodies [110]. In such a scenario, cognitive dysfunction is a common pathological symptom that arises from the high level of lipid peroxidation.

In addition, polyphenols are believed to restore cognition via the modulation of mitochondrial metabolism in the hippocampus region of the brain [111]. Mitochondrial dysfunction can induce the release of NADPH oxidase from the Kreb cycle, thereby accelerating the mitochondrial electron transfer chain. Resultantly, this may raise ROS generation and oxidative stress in the hippocampus [112]. Dysfunctional mitochondria and biogenesis may evince a reduced level of SIRT-1 [113]. SIRT-1 plays a vital role in neuronal plasticity and shield against neuronal degeneration associated with cognitive decline [114]. Under redox conditions, SIRT-1 initiates differentiation in the neural progenitor cells in the astrocytes, thus triggering the accumulation of damaged mitochondrial DNA [115]. This happens as a result of disruption in the integrity of mitochondrial DNA due to a high level of oxidative stress [116]. Thus, the depletion of mitochondrial DNA may activate astrocytes, leading to spongiotic degeneration in the brain parenchyma, as well as severe neurodegeneration, which in due time will manifest as memory loss [117]. However, consumption of polyphenols such as resveratrol [82] and a polyphenol-rich diet comprising various fruits, vegetables, and nuts [90] have proven to improve the level of SIRT-1 drastically in the prefrontal cortex and hippocampus. It has been claimed that a polyphenols-enriched diet can stimulate SRT1460 and SRT218, which in turn may increase the level of SIRT-1 up to five-fold [118]. The increase of SIRT-1 suppresses oxidative stress in the mitochondria via deacetylation of Forkhead box O3 (FOXO3), which may enhance the formation of endogenous antioxidants such as manganese SOD and CAT [119].

In reality, polyphenol metabolites are proven to enter the BBB at a measurable amount across the endothelium [96]. Thus, they would be naturally occurring compounds with an ability to prevent constant injury to the neurons in the brain by providing a shield against the brain neurons [96]. Disruption in the cerebrovascular integrity is one of the most common pathological conditions that lead to cognitive dysfunction in neurological disorders [46,120,121,122]. However, dietary polyphenols can reverse these conditions. Recent discoveries have shown that polyphenols can reduce BBB permeability via various pathways. For instance, quercetin increases the level of SOD [123] and Nrf2 to maintain the permeability and integrity of the BBB by suppressing ROS [124]. Quercetin upregulates the level of claudin-5 and ZO-1 and downregulates the expression of MMP through Wnt/β-catenin downstream mediators to mitigate dysfunctional BBB [125]. As a result, the proinflammatory cytokines, such as NF-κB, TNF-α, and IL-6, will be reduced in the hippocampal region of the brain. At the same time, quercetin impedes the action of E-selectin in the cerebral endothelial cells, therewith preventing monocyte adhesion [126]. Since microglial activation may arise from the transmigration and adhesion of monocytes and enhance cognitive decline [127]; the role of quercetin here could prevent microgliosis, thereby preventing memory loss. Parallelly, polyphenols such as luteolin attenuate cognitive decline [60] through the inhibition of NF-κBp65 translocation, the p38MAPK pathway, and phosphorylated inhibitory κB kinase in the BBB, thus providing a shield against the barrier function of the BBB [128]. Similarly, resveratrol in animal models exhibits its strong antioxidant capacity by suppressing the excessive level of ROS in the BBB and astrocytes and the expression of (NADPH oxidase) 2 and 4 enzymes in the BBB endothelial cells [129].

In addition, administration of certain polyphenols can affect synaptic morphology [130]. For instance, oral administration of 20 mg/kg of resveratrol led to an increase in the length and number of dendritic spines in pyramidal neurons. Since dendrites play a vital role for synaptic function and plasticity, these synaptic morphological changes may have beneficial effects on cognitive function by promoting synaptic plasticity [131]. An intraperitoneal injection of 25 mg/kg of epigallocatechin gallate (EGCG) was found to be effective in restoring dendritic arborization and improving spine maturation in the brain by restoring spine density and maturation [132]. Another mechanism how polyphenols mitigate cognitive impairments is by abating neurotoxins such as excessive level of glutamate [133], and nitric oxide [134] in the brain. Hyperexcitability of glutamate signaling may result in neurodegenerative-associated cognitive decline by inducing neuronal apoptosis [135]. In this, green tea polyphenols enhance the expression of Bcl-2, thereby inhibiting the pore development in the mitochondria by hindering the translocation of Bax from the cytosol into the membrane of mitochondria. Resultantly, activation of caspase 3 and cytochrome C release will be stopped, thereby preventing neuronal apoptosis [133]. Another polyphenol can modulate nitric oxide formation and iNOS expression. Such action in microglia may prevent excessive production of cytokines, in that way preventing injury to the neurons [136]. A similar result was reported during intervening polyphenol-rich blueberry on kainic-acid-infused rat hippocampus. It has been outlined that polyphenol-rich blueberries enhance cognitive behavior by reducing the expression of insulin-like growth factor 1, IL-1β, TNF-α, and NF-κB [137].

### 5.3. The Interplay between Polyphenols, the Gut–Brain Axis, and Cognition

Polyphenols play a vital role in restoring cognition via the signaling pathway involving the gut-brain axis. This is because polyphenols must undergo biotransformation to obtain their metabolites, and this process is accommodated by the gut microbiome. Hence, the gut–brain axis acts as a neuroendocrine system that helps the polyphenols’ metabolites to cross the BBB and exhibit their effects. This may be achieved by various networks involving the enteric nervous system or neuroimmune system [57]. Disruption in the gut–brain axis homeostasis is one cause of cognitive decline [138]. A recent study shows that gut in dysbiosis, a particularly significant reduction in *Eubacterium rectale*—an anti-inflammatory bacterium—with an increased level of *Escherichia coli* and *Shigella*—proinflammatory bacteria—is one of the primary reasons for peripheral inflammation and cognitive decline. This hypothesis was further proven by the presence of abnormal deposition of amyloid in the brain (amyloidosis) [139]. Another study indicates that chronic infection of *Helicobacter pylori* is significantly linked to increased gastric atrophy and cognitive dysfunction. The study proves that *Helicobacter pylori*-infected Alzheimer’s is more susceptible to developing neuroinflammation and cerebrovascular lesions, leading to cognitive impairments [140]. Similarly, excessive level of *Escherichia coli* in the gut microbiota stimulates microgliosis and activates TNF-α, IL-12, and IL-6, eventually resulting in cognitive impairments [141]. In due course, the short-chain fatty acids from microbes mediate the interaction between the bacteria and the microglia cells. When this happens, the bacterial metabolites tend to deviate from colonic mucosa blood circulation, crossing the BBB and inducing neuroinflammation in the brain [142].

**Figure 3:**
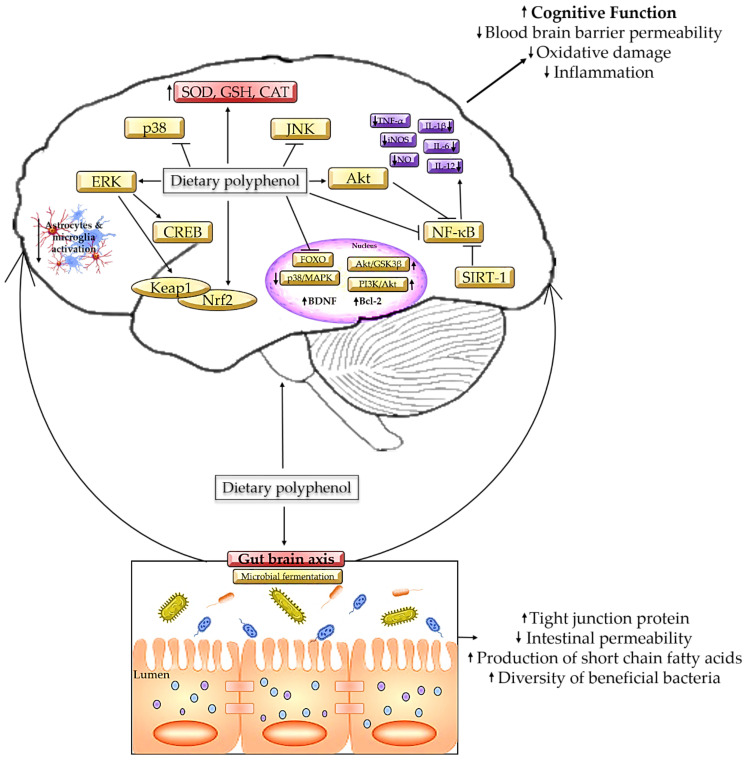
Mechanism on polyphenols in alleviating cognitive decline.

On the contrary, polyphenols could alleviate the damage caused by gut dysbiosis by modifying the colonic microbial composition by influencing bacterial growth and metabolism [8]. *Clostridium perfringens* produces epsilon toxin, which may cleave endothelial cells of the brain, enter the BBB, and induce cellular lesion and glutamate release [143], while *Bacteroides* spp. induce hyperintensity of white matter and hippocampal atrophy and cortical atrophy [144]. In both of these cases, the changes will result in cognitive impairment at some point. However, the high concentration of gallic acid and catechins in tea extract is proven to inhibit the growth of pathogenic bacteria such as *Clostridium perfringens*, *Clostridium difficile,* and *Bacteroides* spp. by stimulating the formation of phenylpropionic acid [145]. Comparably, a dietary intake of curcumin, 700 mg/3 times per day for 4 weeks, successfully eradicates *Helicobacter pylori* colonization [146]. Meanwhile, tea polyphenol epigallocatechin gallates act as pro-oxidants by increasing the level of intracellular oxidative stress to suppress the growth of *Escherichia coli* [147], while gallic acid is proven to hinder *Shigella* spp. by repressing the expression of the *mdoH* and *OpgH* genes [148]. Figure 3 summarizes the mechanism of polyphenols in alleviating cognitive decline.

### 5.4. Polyphenols and Endogenous Antioxidants

The first line of defense against an excessive amount of ROS is initiated by endogenous antioxidants. In these circumstances, SOD, CAT, and GPx are some of the enzymes that provide first-line defense against H_2_O_2_ and superoxide, while aldo-keto reductase, aldheyde dehydrogenase, and glutathione S-transferase (GST) act as a second-line defense against ROS [149]. Upon neutralization by polyphenols, the detoxified products will be cleared from the cells and can be further broken down via the mercapturic acid pathway [150]. Alternatively, a minimum amount of 300 g per day of dietary polyphenol intake maximizes the capacity of endogenous antioxidants via the Nrf2/antioxidant response element (ARE) signaling mechanism and its downstream modulators. Since Nrf2 is the primary regulator for genes responsible for GSH, redox reaction, and antioxidant response; Nrf2 is involved in the modulation of dietary polyphenols and protection against ROS-induced oxidative stress [151]. Upon sensing a rise in oxidative stress, Kelch-like ECH-associated protein 1 (Keap1)—the regulator of Nrf2—restricts the degradation of protein ubiquitination [152]. Subsequently, the accumulation of Nrf2 is increased by transforming phenolics into electrophilic quinones and hydroquinones [153] in the nucleus, thereby increasing the synthesis of endogenous antioxidants of SOD, CAT, and peroxiredoxin [152]. Conversely, a gender-specific study revealed that females tend to have high levels of endogenous antioxidants compared to males. One of the reasons for this could be due to the high level of estrogen in females that modulates the regulatory function of mitochondria [154]. Thus, men need a slightly higher intake of dietary polyphenols compared to women [155] to maximize the protective effects of existing endogenous antioxidants.

## 6. Bioavailability of Dietary Polyphenols

Despite many encouraging findings on the potential effectiveness of dietary polyphenols towards health benefits, the bioavailability of dietary polyphenols remains a main dilemma and has yet to be resolved. Bioavailability can be defined as the proportion of the nutrient that is digested, absorbed, and made usable at the location of the action. The majority of dietary polyphenolic compounds exhibit low bioavailability and are primarily excreted in feces after entering the gut, which results in their poor bioaccessibility [156,157]. As reported by Shivashankara and Acharya (2010), the bioavailability of dietary polyphenols can be ranked in the following order: isoflavones > flavanols > flavanones > flavonols > anthocyanins [158]. High dietary polyphenol intake does not always correspond with high polyphenol bioavailability [159]. The bioavailability of dietary polyphenols can vary depending on several factors, including the type of dietary polyphenol, the food matrix it is found in, food processing, digestive enzymes, the microenvironment of the intestine, metabolism rate, and gut microbiota composition. The main factor that has been identified as causing poor bioavailability of dietary polyphenols is a first-pass metabolism that involves phase II conjugation, such as like methylation, glucuronidation, or sulfonation [160]. This metabolic reaction will convert dietary polyphenol into hydrophilic conjugates known as glucuronides and sulfates, which are then easily eliminated via urine [160,161,162].

Biotransformations promoted by the gut microbiota can have a significant impact on the bioavailability of polyphenols. The gut microbiota can transform a polyphenol through enzymatic reactions, such as fermentation, oxidation, reduction, and hydrolysis. These biotransformations can alter the chemical structure of these compounds, affecting their solubility, stability, and absorption. One of the factors that can influence the level of biotransformations of polyphenols in the gut is the individual diversity of intestinal microbiota. For instance, deglycosylation can be performed by a wide array of gut microbial species and genera; others require particular enzymes that are only expressed by a given species or strain. Deglycosylation will produce an aglycone form of the polyphenol which reduces both the bioaccessibility and bioavailability of phytochemicals [21,163,164].

## 7. Dosage and Safety Concerns of Dietary Polyphenols

The effective dosage of dietary polyphenols can vary depending on the specific compound. Some studies have suggested that lower doses of certain polyphenols, such as resveratrol, may be more effective than higher doses at providing neuroprotection against cognitive decline [165,166]. When evaluating the therapeutic potential of dietary polyphenols, CNS (central nervous system) penetrability is an important consideration. The ability of a compound to penetrate the CNS can be influenced by factors such as molecular weight, lipophilicity, and the presence of polar groups in the compound. Some polyphenols, such as curcumin, have poor CNS penetrability due to their low lipophilicity and high molecular weight [167]. In contrast, others—such as EGCG—have been shown to readily cross the BBB even in very low doses [168]. The amount of dietary polyphenol intake influences the beneficial effect it could exhibit. A study published by Perry et al. (2018) indicates that daily consumption of 500 mg of polyphenol could help to reduce oxidative stress, while 1000 mg/day is enough to suppress inflammation. Similarly, up to 1500 mg of dietary polyphenol intake could enhance mitochondrial synthesis [169]. Based on epidemiological data collected, it has been suggested that a mean consumption of 900 mg/day is the daily recommended dosage [170]. However, the minimal dosage requirements could differ for each individual based on underlying disease condition, daily lifestyle, geographical location, or source of polyphenol. For example, for obese subjects, a daily dosage of not more than 1200 mg is recommended daily [171], while the safe dose for pregnant women would be 500 mg/day [172]. Conversely, the dosage effects of polyphenols may depend on the age and strain of the animals. For example, one study found that chronic treatment with resveratrol improved cognitive function in aged rats [173] but not in young mice [174]. Similarly, a study on the effects of blueberry extract on cognitive function found that supplementation improved memory in aged mice [175] but had no effect on young mice. In terms of strain differences, a study comparing the effects of green tea extract on cognitive function in two different strains of mice found that the extract improved memory in SAMP8 but not in the SAMR1 [176]. It is important to know the recommended dosage, as excessive consumption of polyphenols could induce adverse effects.

A toxicity evaluation shows that continuous intake of quercetin for 2 years at 40,000 ppm daily has induced chronic nephropathy and carcinogenic symptoms in the kidney in Fischer 344 male rats. Thus, from the documented symptoms, quercetin has been termed a genotoxic chemical [177]. Similarly, a daily intake of 40 mg of quercetin for 50 weeks decreased the life span of male LACA mice, while the same dose in female LACA mice increased the life expectancy [178]. Therewithal, a diet supplement containing 2% of caffeic acid in Fischer 344 rats and C57BL/6N x C3H/HeN F1 mice resulted in aggressive carcinogenic development in the abdominal cavity and renal tubule [179]. One of the possible underlying causes for this could be due to the ability of quercetin to inhibit catechol O-methyltransferase (COMT) [180] which in turn may trigger redox reaction in catecholestrogens, eventually accelerating the progression of estradiol-induced carcinogenesis [181]. Similarly, 1% of green tea catechins diet supplements for 33 weeks resulted in cancer cell proliferation in Fischer 344 male rats [182]. Another study theorizes that polyphenol-rich tea consumption positively correlates with iron deficiency in women [183], while proanthocyanidins escalate the level of endogenous digestive proteins, such as protease, lipase, and biliary acid [184]. Additionally, grape seed polyphenolic extract exhibited pro-oxidants and deleterious effects in cells [185] and interfered with the pharmacokinetics and bioavailability of certain drugs (benzodiazepines and terfenadine) [186].

In toto, while some polyphenols have been shown to have potential health benefits, such as antioxidant and anti-inflammatory effects in restoring cognition as discussed in this study, others may have harmful effects (carcinogenic or genotoxic) at high doses [181]. One challenge in evaluating the potential toxicity of polyphenols is the variability in the types and amounts found in different foods. Additionally, some polyphenols may be transformed by gut bacteria into metabolites that have different effects on the body than the original compound [187]. Although there is some evidence to suggest that high doses of certain polyphenols, such as catechins found in green tea, may have toxic effects on the liver in very rare cases [188], these effects have typically been observed in studies using very high doses that are unlikely to be encountered in normal dietary intake. Moderate long-term use of certain polyphenols, such as resveratrol found in grapes and red wine, has been associated with potential health benefits, such as improved memory function as described in Table 1. However, the evidence for these benefits is still limited, and more research is needed to determine optimal doses and potential risks.

## 8. Conclusions

In conclusion, there has been growing interest in the potential of dietary polyphenols to protect against cognitive decline. The reviewed animal studies have provided strong evidence that polyphenol intake can reverse cognitive dysfunction via various pathways, including reducing neuroinflammation, oxidative stress, and Aβ deposition, improving mitochondrial function and synaptic plasticity and modulating gut microbiota. However, it is important to note that excessive intake of polyphenols can also have adverse effects, such as gastrointestinal discomfort, liver toxicity, and interference with nutrient absorption. Therefore, appropriate dosage taking into account individual factors, such as gender, underlying conditions, and lifestyle, is crucial. Hence, while the animal experiments provide a strong foundation for the therapeutic potential of dietary polyphenols in cognitive decline, more research is needed to determine the optimal dosage and long-term effects of polyphenol intake in humans. It is also important to consider individual factors when recommending polyphenol intake for memory enhancement. Nevertheless, the findings suggest that dietary polyphenols hold promise as a safe and effective therapeutic strategy for cognitive impairment, especially when integrated with other lifestyle changes.

## 9. Limitation

One limitation of this study could be the sex of the animal models used in the study. Since most of the studies use male animal models, the outcomes may not accurately reflect the effects of dietary polyphenols on female subjects. This may limit the generalizability of the findings to female subjects, as there might be evidence to suggest that the neuroprotective effects of polyphenols may differ between males and females due to differences in sex hormones and other biological factors. Thus, future studies should aim to include both male and female animal models to better understand the sex-specific effects of polyphenols on brain health. This is to avoid potential biases and highlight the importance of considering sex differences in future studies. Additionally, although the selected papers may suggest that polyphenols compounds have a protective effect against neuronal death, the manuscripts may not sufficiently address the concentrations at which these effects occur. This could be seen as a limitation of the study, as it may not provide a comprehensive understanding of how the compounds function in vivo. Another limitation is that animal models may not always translate directly to human subjects. The effects of dietary polyphenols on cognitive decline and neuroprotection in humans may be different than those observed in animal models. Additionally, the specific type and dose of polyphenols used in animal models may not accurately reflect the polyphenol intake of humans. Human diets are more complex and may contain a wider variety of polyphenols than those used in animal studies. Finally, it is important to note that the effects of polyphenols on cognitive decline and neuroprotection are likely to be influenced by a variety of factors beyond diet, including genetics, lifestyle, and environmental factors.

## Figures and Tables

**Table 1:** Natural polyphenols for neuroprotection in cognitive impairments.

Author	Intervention	Animal Model	Dosage, and Duration	Treatment Mode	Behavioral Test	Findings
Liu et al., 2013 [59]	Mangiferin	10-week-old male Sprague Dawley rats injected with streptozotocin (STZ)	15, 30, and 60 mg/kg/day for 9 weeks	Oral gavage	- Morris water maze	- The time in mean escape latency had decreased. - The time spent at the targeted quadrant had increased. - The number crossing the platform had increased. - The level of advanced glycation end-product (AGE), the receptor for AGE (RAGE), and malondialdehyde (MDA) levels in the hippocampus had decreased. - The level of glyoxalase 1 (Glo-1), GSH, and tumor necrosis factor (TNF)-α in the hippocampus had increased. - The level of interleukin-1β (IL-1β) in the hippocampus had decreased.
Liu et al., 2013 [60]	Luteolin	10-to-12-week-old male Sprague Dawley rats injected with STZ	50, and 100 mg/kg/day for 8 weeks	Oral gavage	- Probe trial - Morris water maze	- The transfer latency had decreased. - The time spent at the targeted quadrant had increased. - The number crossing the platform had increased. - The number of surviving neurons in the hippocampus had increased. - The activity of cholinesterase in the cortex and hippocampus had decreased. - The level of MDA in the hippocampus had decreased. - The levels of SOD, GSH, and CAT in the cerebral cortex and hippocampus had increased.
Grossi et al., 2013 [61]	Oleuropein Aglycone	1.5-to-4-month-old transgenic CRND8 mice encoding a double-mutant of APP695	50 mg/kg/day for 8 weeks	Diet	- Step down inhibitory avoidance task - Object recognition test	- The discrimination score had increased. - The time spent on the novel object had increased. - The level of amyloid beta (Aβ) plaque area and number in the cerebral cortex and hippocampus had decreased. - The expression of Beclin-1 in the soma, perikarya, and dendrites in CA1, CA3, and dentate gyrus had increased. - The amyloid deposition and increased level of plaque clearance in the hippocampal region had decreased.
Cheng et al., 2014 [62]	Apple (Ralls) polyphenol	7-week-old male Wistar rats	200 mg/kg/day for 10 weeks	Diet	- Step down inhibitory avoidance task - Morris water maze	- The time in mean escape latency had decreased. - The time spent at the targeted quadrant had increased. - The number crossing the platform had increased. - The activity level of acetylcholinesterase (AChE) and creatine kinase as well as that of ATP synthesis in the cerebral cortex had increased. - The levels of Aβ_42_ and MDA in the forebrain had decreased. - The levels of SOD and CAT in the brain had increased. - The levels of blood congestion, neuron disruption, and vacuolization had decreased.
Xia et al., 2014 [63]	Quercetin	5-week-old male Chinese Kunming mice	0.005% and 0.01% (*w*/*w*) for 12 weeks	Diet	- Morris water maze	- The escape latency period had increased. - The time spent at the targeted quadrant had increased. - The level of MDA in the hippocampus had decreased. - The level of SOD and total antioxidant capacity (TAC) in the hippocampus had increased. - The levels of brain-derived neurotrophic factor (BDNF), phosphoinositide 3 kinases (PI3K), Akt, and Nrf2 in the hippocampus had increased.
Xia et al., 2014 [64]	Salvianolic acid B	4-to-6-week-old Male C57BL/6 mice	14 mg/kg/day for 7 weeks	Drinking water	- Morris water maze	- The escape latency period had increased. - The time spent at the targeted quadrant had increased. - The levels of BDNF, SOD, glutathione peroxidase (GPx), and thiobarbituric acid reactive substances (TBARS) in the hippocampus had increased. - The levels of TNF-α, IL-6 and monocyte chemotactic protein-1 (MCP-1) levels in serum and the hippocampus had decreased.
Zhao et al., 2015 [65]	Grape seed polyphenol extract, concord grape juice, resveratrol, quercetin, and malvidin-glucoside	C57BL6/J mice	0.02, 0.2, 2, 20, or 200 mg/kg/day or malvidin-3-O-glucoside 0.05, 0.5, 5, and 50 mg/kg/day for 10 days and 0.2 mg/kg/day and malvidin-3-O-glucoside 5 mg/kg/day for 6 weeks	Drinking water	- Contextual fear conditioning test	- The contextual memory had improved. - The phosphorylation of CREB on Ser133 in the hippocampus had increased.
Souza et al., 2015 [66]	Chrysin	3- and-20-month-old male Swiss albino mice	1 or 10 mg/kg/day for 60 days	Oral gavage	- Open field test - Morris water maze	- The escape latency period had increased. - The increased time spent at the targeted quadrant had increased. - The level of reactive species in the prefrontal cortex and hippocampus had decreased. - The levels of CAT, SOD, and GPx in the prefrontal cortex and hippocampus had increased. - The levels of Na^+^, K^+^, and -ATPase activity and BDNF in the hippocampus had increased.
Kuo et al., 2015 [67]	Prunus Salicina	4-week-old C57BL/6 mice	2% or 5% for 5 months	Diet	- Morris water maze	- The level of TBARS in the serum had increased. - The escape latency period had increased. - The time spent at the targeted quadrant had increased. - The level of β secretase 1 (BACE1) and Aβ in the cortex and hippocampus had decreased.
Bensalem et al., 2016 [68]	Polyphenol-rich extract from grape and blueberry	6-week- and 6-month-old male C57Bl/6J	500 mg/kg/day for 8 weeks	Diet	- Morris water maze	- The escape latency period had increased. - The time spent at the targeted quadrant had increased. - The level of extracellular signal-regulated kinases (ERK) 1 in the hippocampus had decreased. - The levels of calmodulin-dependent protein kinase II (CaMKII), BDNF, and nerve growth factor (NGF) in the hippocampus had increased.
Chou et al., 2016 [69]	Vitis vinifera	66-week-old male Wistar rats	3% or 6% for 12 weeks	Diet	- Morris water maze	- The escape latency period had increased. - The time spent at the targeted quadrant had increased. - The number crossing the platform had increased. - The level of α-secretase in the cortex and hippocampus had increased. - The level of BACE, and Aβ deposition in the cortex and hippocampus had decreased. - The level of GSH/GSSG in the hippocampus had increased.
Dal-Pan et al., 2016 [70]	Neurophenols consortium extract	12-month-old homozygous 3xTg-AD	500 or 25,000 mg/kg/day for 4 months	Diet	- Open field test - Novel object recognition test (NORT)	- The level of recognition index in NORT had increased. - The level of BDNF in the cortex had increased.
Tian et al., 2016 [71]	Resveratrol	10-to-12 week-old male Sprague Dawley rats	10 or 20 mg/kg/day for 8 weeks	Oral gavage	- Morris water maze - Memory consolidation test	- The transfer latency period had decreased. - The mean escape latency period had decreased. - The time spent at the targeted quadrant had increased. - The number crossing the platform had increased. - The levels of MDA, TNF-α, and IL-1β in the hippocampus had decreased. - The level of GSH in the hippocampus had increased. - The level of synaptic protein (SYN and GAP-43) in the hippocampus had increased.
Wang et al., 2017 [72]	Teasaponin	8-week-old male C57BL/6J mice	0.5% for 6 weeks	Diet	- NORT	- The levels of TNF-α, IL-6, IL-1β, TLR4, and MyD88 in the hippocampus had decreased. - The levels of p-JNK and NF-κB in the hippocampus had decreased. - The levels of Iba-1 and GFAP in the hippocampus had decreased. - The level of BDNF in the hippocampus had increased. - The discrimination index in NORT had increased.
Sawmiller et al., 2017 [73]	Pyrroloquinolinequinone	3- and 6-month-old female 5XFAD transgenic mice	1.6 or 1.8 mg/kg/day for 12 weeks	Diet	- Rotarod test - Open field test - Elevated plus maze (EPM) - Morris water maze - Y maze - Fear conditioning test	- The time spent at the rod had increased. - The total distance travelled, and average speed in EPM had increased. - The escape latency period had decreased. - The time spent at the targeted quadrant had increased. - The number crossing the platform had increased. - High level of spontaneous alternation in the Y maze was spotted. - The freezing in the context test had increased. - The level of amyloid plaques in the hippocampus, entorhinal, and retrosplenial cortex had decreased. - The level of reactive oxygen species (ROS) and mitochondrial membrane potential (MMP) hyperpolarization in the brain had decreased.
Abhijit et al., 2017 [74]	Grape Seed Proanthocyanidin	4- and 18-month-old male Wistar rats	400 mg/kg/day for 120 days	Oral gavage	- T maze	- The level of correct choices in the T maze had increased. - The activity of AChE and M1 AChR in the CA1 of the hippocampus had increased.
Jinbo et al., 2017 [75]	Resveratrol	10-week-old male senescence-accelerated mouse resistant 1 (SAMR1) and SAMP8 mice	30 mg/kg/day for 24 weeks	Diet	- Morris water maze	- The time spent at the targeted quadrant had increased. - The number crossing the platform had increased. - The levels of Aβ42, BACE1, tau phosphorylation, GFAP, and phosphorylated NF-κB p65 in the hippocampus had decreased. - The level of phosphorylated cAMP-response-element-binding protein (CREB) in the hippocampus had increased.
Fragua et al., 2017 [76]	Blueberry and grape extract	8-to-14.5-year-old male and female beagle dogs	240 or 480 ppm for 75 days	Diet	- Delayed non-matching to position testing	- The levels of SOD and Nrf2 in the hippocampus had increased. - The working memory had improved.
Gomaa et al., 2018 [77]	Boswellia serrata gum	8 to 12-months- old male Wistar rats	200, 300 and 400 mg/kg/day for 8 weeks	Oral gavage	- Passive avoidance task - Morris water maze	- The step-through latency had increased. - The time spent at the targeted quadrant had increased. - The number crossing the platform had increased. - The levels of Aβ deposits, phosphorylated tau, caspase 3, and glycogen synthase kinase-3β (GSK-3β) activity in the hippocampus had decreased. - The level of cholinesterase in the hippocampus had increased. - The levels of TNF-α, IL-1β, IL-6, and MDA in the hippocampus had decreased. - The levels of GluR1, NR1, NR2 A, and NR2B in the hippocampus had increased.
Sharma et al., 2018 [78]	Apigenin	3-month-old Swiss albino mice	10 or 20 mg/kg/day for 20 days	Oral gavage	- T maze - EPM - Tail suspension test	- The spontaneous alternation in the T maze had increased. - The anxiety index in EPM had decreased. - The immobility time in the tail suspension test had decreased. - The levels of BDNF, CREB, and serotonin in the hippocampus had increased.
Sanna et al., 2018 [79]	Grape seed proanthocyanidin	Adult male Wistar rats induced with STZ	75 mg/kg/day for 16 weeks	Oral gavage	- T maze	- The level of correct choices made in the T maze had increased. - The number of surviving neurons in the prefrontal cortex had increased. - The well-defined cristae and membranes of mitochondria had increased. - The level of Bcl-2 in the prefrontal cortex had increased. - The level of Bax in the prefrontal cortex had decreased.
Zhang et al., 2018 [80]	Oleanolic acid	7-to-8-week-old male Sprague Dawley rat	5 mg/kg/day for 10 days	Intraperitoneal injection	- Morris water maze	- The time in mean escape latency had increased. - The time spent at the targeted quadrant had increased. - The number crossing the platform had increased.
Bensalem et al., 2018 [81]	Polyphenol-rich extract from grape and blueberry	6-week- and 16-month-old male C57Bl/6J mice	500 or 600 mg/kg/day for 14 weeks	Diet	- NORT - Morris water maze	- The duration spent at the novel object in NORT had increased. - The time in mean escape latency had decreased. - The time spent at the targeted quadrant had increased. - The number crossing the platform had increased. - The levels of NGF and newly formed neurons in the hippocampus had increased.
Qi et al., 2018 [82]	Resveratrol	Male Kunming mice	0.02 or 0.2 mg/kg/day for 10 days	Intramuscular injection	- Y maze - Morris water maze	- The spontaneous alternation in the Y maze had increased. - The time in mean escape latency had decreased. - The time spent at the targeted quadrant had increased. - The number crossing the platform had increased. - The level of SIRT-1, AMPK and PGC-1α in the prefrontal cortex and hippocampus had increased. - The levels of NF-κB, IL-1β and NLRP3 in the prefrontal cortex and hippocampus had decreased.
Yu et al., 2018 [83]	Gallic acid	4-month- and 9-month-old male APP/PS1 mice	30 mg/kg/day for 30 days	Oral gavage	- Open field test - Morris water maze - Y maze - NORT	- The spontaneous alternation in the Y maze was spotted. - The time in mean escape latency had decreased. - The time spent at the targeted quadrant had increased. - The number crossing the platform had increased. - The duration spent at the novel object in NORT had increased. - The levels of PSD95 and synaptophysin in the hippocampus had increased. - The level of GFAP in the hippocampus had decreased. - The level of dendritic spines in the hippocampal neurons had increased. - The level of Aβ plaque size and Aβ_1–42_ aggregation in the brain had decreased.
González-Granillo et al., 2022 [84]	Curcumin	18-month-old male C57BL/6 mice	20 mg/kg for 5 days per week for 2 months	Intraperitoneal injection	- Moris water maze - NORT	- The duration of escape latency had decreased. - The total exploration time at the novel object in NORT had increased. - The dendritic arbor, total dendritic length, and spine density had increased. - The expression of synaptophysin in the prefrontal cortex and hippocampus had increased. - The expression of actin in the prefrontal cortex had increased. - The expression of CA3 in the hippocampus had increased. - The expression of astrocytes in the CA1 and CA3 regions of the hippocampus had decreased.
Ishida et al., 2019 [85]	Coffee extract	5-week-old male APP/PS2 mice	1% for 5 months	Diet	- NORT - Morris water maze - Step-through passive avoidance task	- The total exploration time at the novel object in NORT had increased. - The duration of escape latency had decreased. - The time spent at the targeted quadrant had increased. - The latency time in the step-through passive avoidance task had increased. - The Aβ plaque deposition and APP mRNA in the hippocampus had decreased.
Izquierdo et al., 2019 [86]	Resveratrol	Male and female SAMP8 mice	1 g/kg/day for 2 months	Diet	- NORT - Morris water maze	- The total exploration time at the novel object in NORT had increased. - The duration of escape latency had decreased. - The time spent at the targeted quadrant had increased. - The level of Nrf2 in the hippocampus had increased. - The level of NF-κB in the hippocampus had decreased. - The expression of Beclin-1 and phosphorylated AMPK in the hippocampus had increased. - The level of phosphorylated mTOR in the hippocampus had decreased.
Choi et al., 2019 [87]	Vigna angularis	5-week-old male C57BL/6J mice	100 and 200 mg/kg/day for 4 weeks	Oral gavage	- T maze - NORT - Morris water maze	- The total exploration time at the novel object in NORT had increased. - The duration of escape latency had decreased. - The time spent at the targeted quadrant had increased. - The level of correct choices in the T maze had increased.
Lee et al., 2020 [88]	Chlorogenic acid	6-month-old male Mongolian gerbils	7.5, 15, and 30 mg/kg/day for 5 days	Intraperitoneal injection	- Passive avoidance task - 8 arm radial maze (8-ARM)	- The number of errors in 8-ARM had decreased. - The latency time in the passive avoidance task had increased. - The number of neurons or pyramidal cells in the CA1 region of the hippocampus had increased. - The levels of SOD2, IL-13, and IL-4 in the CA1 region of the hippocampus had increased. - The levels of IL-2 and TNF-α in the CA1 region of the hippocampus had decreased.
Ishida et al., 2020 [89]	5-Caffeoylquinic acid	10-week-old male APP/PS2 mice	1% for 4 months	Diet	- Y maze - NORT	- The level of percentage of alteration in the Y maze had increased. - The ability to discriminate familiar and novel objects in NORT had increased. - The level of Aβ plaque deposition in the cerebral cortex and hippocampus had decreased. - The level of neuronal loss in the CA1, CA2, and CA3 regions of the hippocampus had decreased. - The level of perivascular aquaporin 4 (AQP4) and low-density lipoprotein receptor-related protein 1 (LRP1) in the hippocampus had increased.
Ramis et al., 2021 [90]	Polyphenol-Enriched Diet	Male Wistar rats	30 g/rat/day for 20 weeks	Diet (Pellet)	- 8 arm radial maze (8-ARM) - NORT - Rotarod Test - Barnes holeboard maze	- The ability to locate targets more quickly in the Barnes maze test had increased. - The explorative time in the novel object in NORT had increased. - The levels of 5-HTP, 5-HIAA, and SIRT-1 in the hippocampus had increased.
Yamamoto et al., 2021 [91]	Rosmarinic acid	8-week-old male 3 × Tg-AD mice	0.5% for 8 months	Diet	- Y maze - NORT	- The alternation ratio in the Y maze had increased. - The recognition index of the novel object in NORT had increased. - The level of Aβ plaques in the CA1 region of the hippocampus had decreased. - The downregulation of the MAPK/JNK3 signaling pathway was noted. - The inhibition of the JNK signaling pathway was noted. - The levels of Ccl5 and Cxcl13 in the hippocampus had decreased. - The level of TLR2 in the cerebral cortex had decreased. - The level of TLR4 in the hypothalamus had decreased.
Zhang et al., 2021 [92]	Tea polyphenols	3-to-4-month-old female Sprague Dawley	75, 150, and 300 mg/kg/day for 12 weeks	Intragastric administration	- Open field test - Morris water maze	- The duration of escape latency had decreased. - The time spent at the targeted quadrant had increased. - The level of Aβ_1–42_ in the hippocampus had decreased. - The levels of glucose transporters (GLUT) 1, GLUT3, and O-GlcNAc glycosylation of tau protein in the hippocampus had increased. - The level of tau protein phosphorylation in the hippocampus had decreased. - The integrity of mitochondria in the hippocampal neuron had increased.
Yang et al., 2021 [93]	Tea polyphenols and proanthocyanidins	7-to-8-week-old female Sprague Dawley rats	100 and 150 mg/kg/day for 12 weeks	Intragastric administration	- Morris water maze	- The time in mean escape latency had decreased. - The time spent at the targeted quadrant had increased. - The number crossing the platform had increased. - The long-term potentiation (LTP) had increased. - The dendrites lengths, branches, and spine in the hippocampal neurons had increased. - The levels of PSD95, GluR1, and GluR2 in the hippocampus had increased. - The levels of Aβ_1–42_ and ionized-calcium-binding adaptor molecule-1 (Iba-1) in the hippocampus had decreased. - The level of p38 and TNFR2 genes in the hippocampus had increased. - The levels of TNF-α and TNFR1 genes in the hippocampus had decreased.
Mikami et al., 2021 [94]	Olive oil	10-week-old male C57BL/6J mice	1 g/1000 high-fat diet for 10 weeks	Diet	- Y maze - forced swimming test - Sucrose preference test (SPT)	- The spontaneous alterations in the Y maze had increased. - The sucrose preference ratio SPT had increased. - The immobility time in a forced swimming test had decreased. - The level of lipid peroxide in the hippocampus had decreased. - The levels of total radical trapping antioxidant parameter (TRAP), PGC-1α, and SIRT-1 mRNA and SOD in the hippocampus had increased.
Kang et al., 2022 [95]	Epicatechin	8-week-old male C57BL/6J mice	2 or 20 mg/kg/day for 24 weeks	Diet	- Open field test - NORT - Morris water maze	- The time spent in the central zone and total distance travelled in an open field test had increased. - The time spent at the targeted quadrant had increased. - The number crossing the platform had increased. - The levels of BDNF, glucocorticoid, and mineralocorticoid receptors in the hippocampus had increased. - The level of 11 β-HSD1 in the hippocampus had decreased.

## Data Availability

Not applicable.
